# Supplementary material for: Allosteric nanobodies to study the interactions between SOS1 and RAS
Source: Nat Commun. 2024 Jul 23;15:6214. doi: 10.1038/s41467-024-50349-2 (PMC11266648; doi:10.1038/s41467-024-50349-2)
Supplement: Supplementary file 1 — Supplementary Information [file 41467_2024_50349_MOESM1_ESM.pdf]

Supplementary Information

Allosteric nanobodies to study the interactions between SOS1 and RAS

Baptiste Fischer[1,2], Tomasz Uchański[3,4], Aidana Sheryazdanova[5,6], Simon Gonzalez[7], Alexander N. Volkov[3,8], Elke Brosens[3,4], Thomas Zögg[3,4], Valentina Kalichuk[3,4], Steven Ballet[7], Wim Versées[3,4], Anna A. Sablina[5,6], Els Pardon[3,4], Alexandre Wohlkönig[3,4] and Jan Steyaert[3,4*]

[1]Université de Bordeaux, CNRS, Bordeaux INP, CBMN, UMR 5248, F-33600 Pessac, France
[2]Structural Biology of Biofilms group, European Institute of Chemistry and Biology (IECB), 2 rue Robert Escarpit, F-33600 Pessac, France
[3]VIB-VUB Center for Structural Biology, VIB, Pleinlaan 2, 1050 Brussels, Belgium
[4]Structural Biology Brussels, Vrije Universiteit Brussel, Pleinlaan 2, 1050 Brussels, Belgium
[5]VIB-KU Leuven Center for Cancer Biology, VIB, Herestraat 49, 3000 Leuven, Belgium
[6]Department of Oncology, KU Leuven, Herestraat 49, 3000 Leuven, Belgium
[7]Research Group of Organic Chemistry, Vrije Universiteit Brussel, Pleinlaan 2, 1050 Brussels, Belgium
[8]Jean Jeener NMR Centre, VUB, Brussels, Belgium

***Correspondence:** jan.steyaert@vub.be

Content :

Supplementary Figures 1-11
Supplementary Table 1
Supplementary References

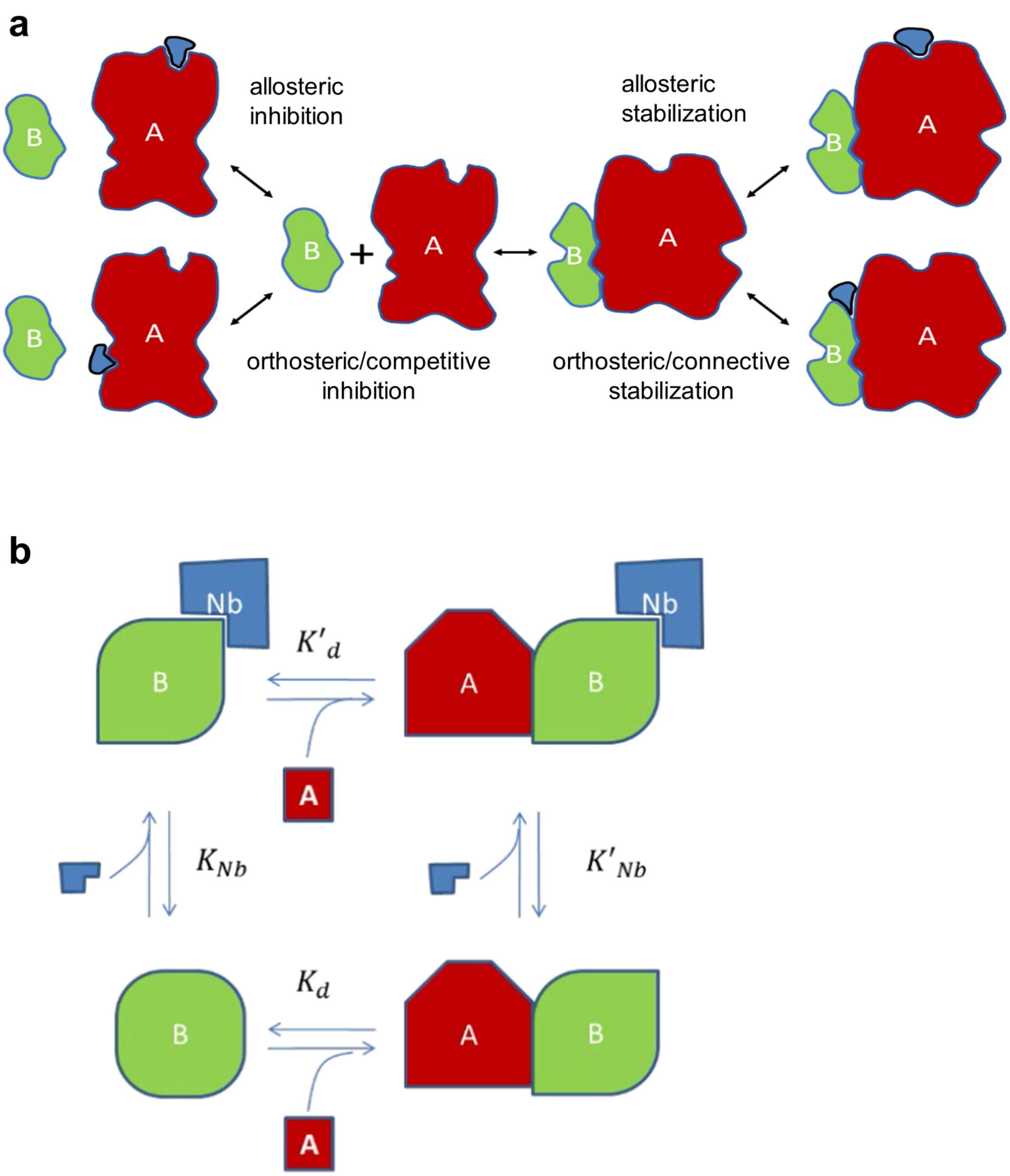

Supplementary Figure 1. Modulation of protein-protein interactions by ligands.
a. Depending on the binding mode of a chemical or biological compound (blue), PPIs can be modulated in different ways. **b.** Thermodynamics imply that any Nb that preferentially binds an allosteric or connective conformational epitope characteristic for the complex, will stabilize the complex A•B proportionally: $K_{Nb} \times K'_{d} = K'_{Nb} \times K_{d}$ or $K_{Nb}/K'_{Nb} = K_{d}/K'_{d}$.

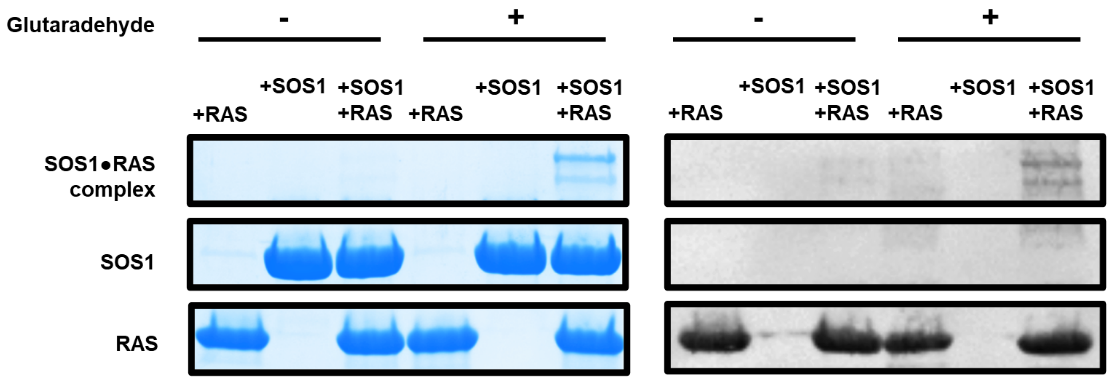

Supplementary Figure 2. Crosslinking of the SOS1•RAS complex monitored on SDS-PAGE stained by Coomassie blue (left) and by immunodetection of the His-tag on RAS (right). RAS (with His-tag) and SOS1 (without His-tag) were purified to homogeneity and mixed at a 2:1 molar ratio with (+) and without (-) glutaraldehyde (0.001% final concentration) for 1 h at room temperature. Glutaraldehyde induced the formation of two bands migrating at molecular weights corresponding to the binary SOS1•RAS complex and the ternary RAS•SOS1•RAS complex.

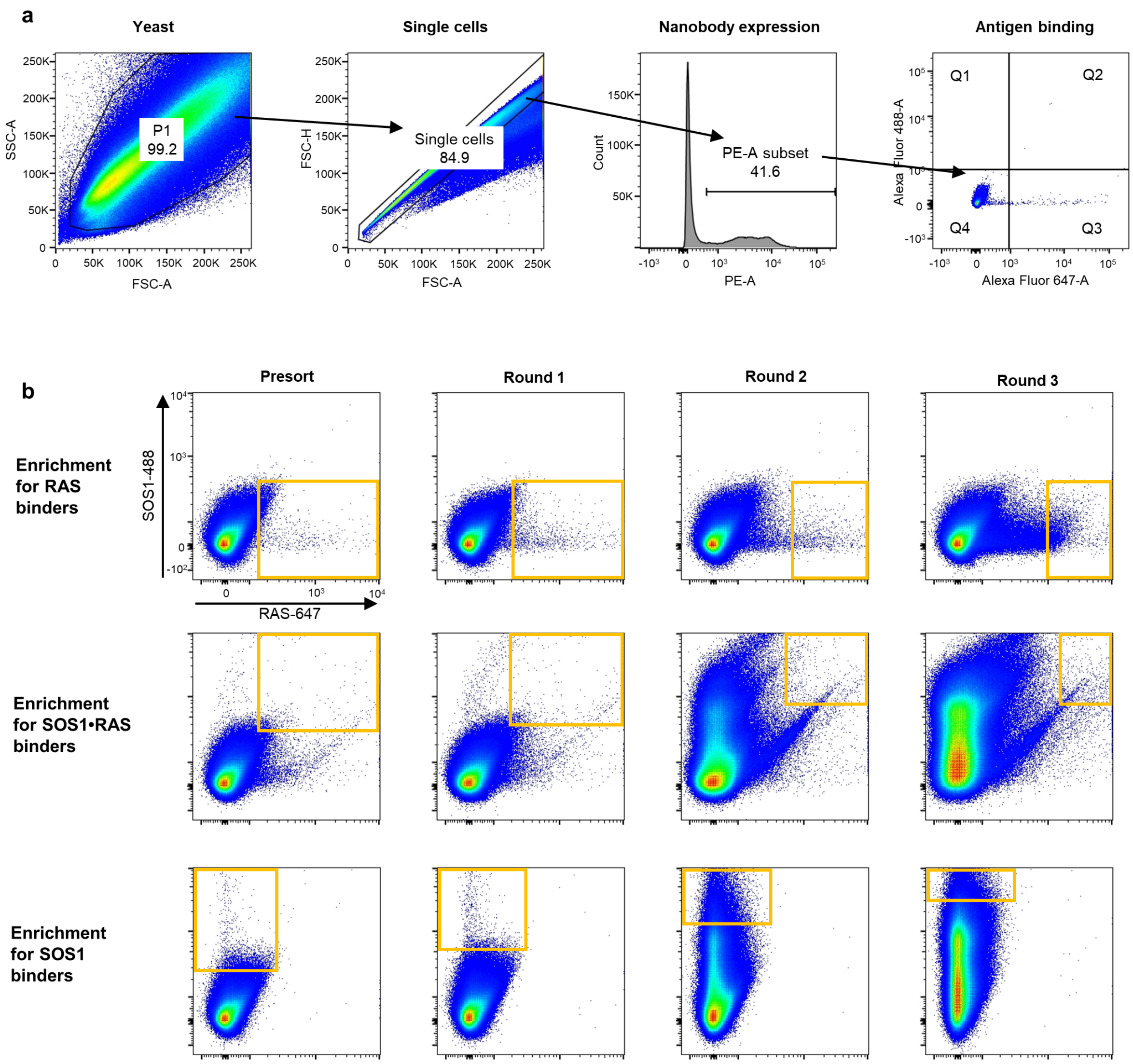

Supplementary Figure 3. FACS gating strategy for the sorting of yeast cells displaying a nanobody that binds the SOS1•RAS complex or its protomers. a. Yeast cells displaying a Nb on the surface were stained with 2 µM CoA-Alexa547 and incubated with 100 nM of the fluorescent proteins. SOS1 was labeled with Alexa488 and RAS was labeled with Alexa647. For sorting, we first gated on the side scattering (SSC-A) and forward scattering (FSC-A) to recover single cells. Next, we gated on Alexa547 fluorescent cells to recover cells expressing a Nb on the surface. Finally, cells were analyzed for the fluorescence at 488 or 647 to discriminate SOS1-488 binders (Q1) from RAS-647 binders (Q3) from binders to the complex (Q2). **b.** Stepwise enrichment of yeast cells displaying Nbs in three rounds of Disco followed by FACS. Each dot represents two fluorescent signals of a separate yeast cell of the (sub) library. The x- and y-axis are the relative measure of the fluorescence of RAS-647 (x-axis) and SOS1-488 (y-axis). Yeast with the desired fluorescence (yellow square) were sorted and cultured for the next round of selection.

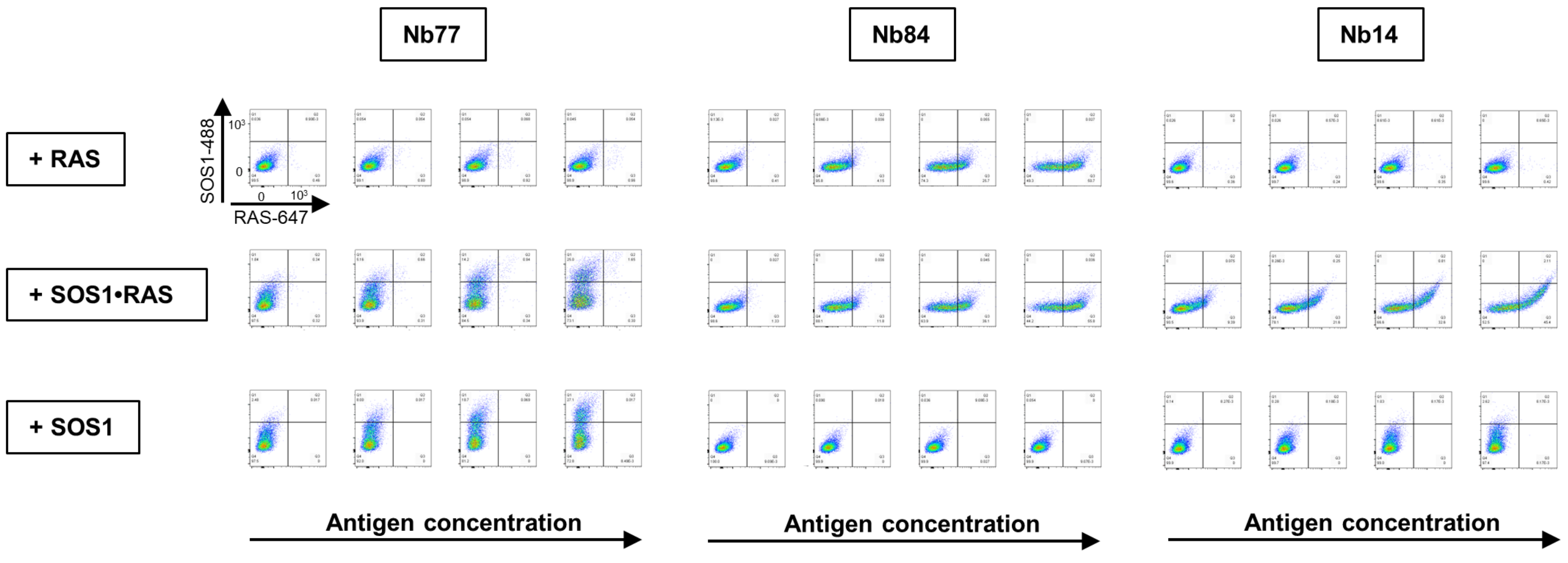

Supplementary Figure 4. Analysis of the binding properties of nanobodies from three representative classes by flow cytometry: Nb84 was recovered from Q3, Nb14 was recovered from Q2 and Nb77 was recovered from Q1. Yeast clones displaying these Nbs were cultured and incubated with increasing concentrations (6, 12, 25, 50 nM) of fluorescently labeled SOS1-488 or RAS-647, separately or combined, to analyze the binding properties of these Nbs by flow cytometry.

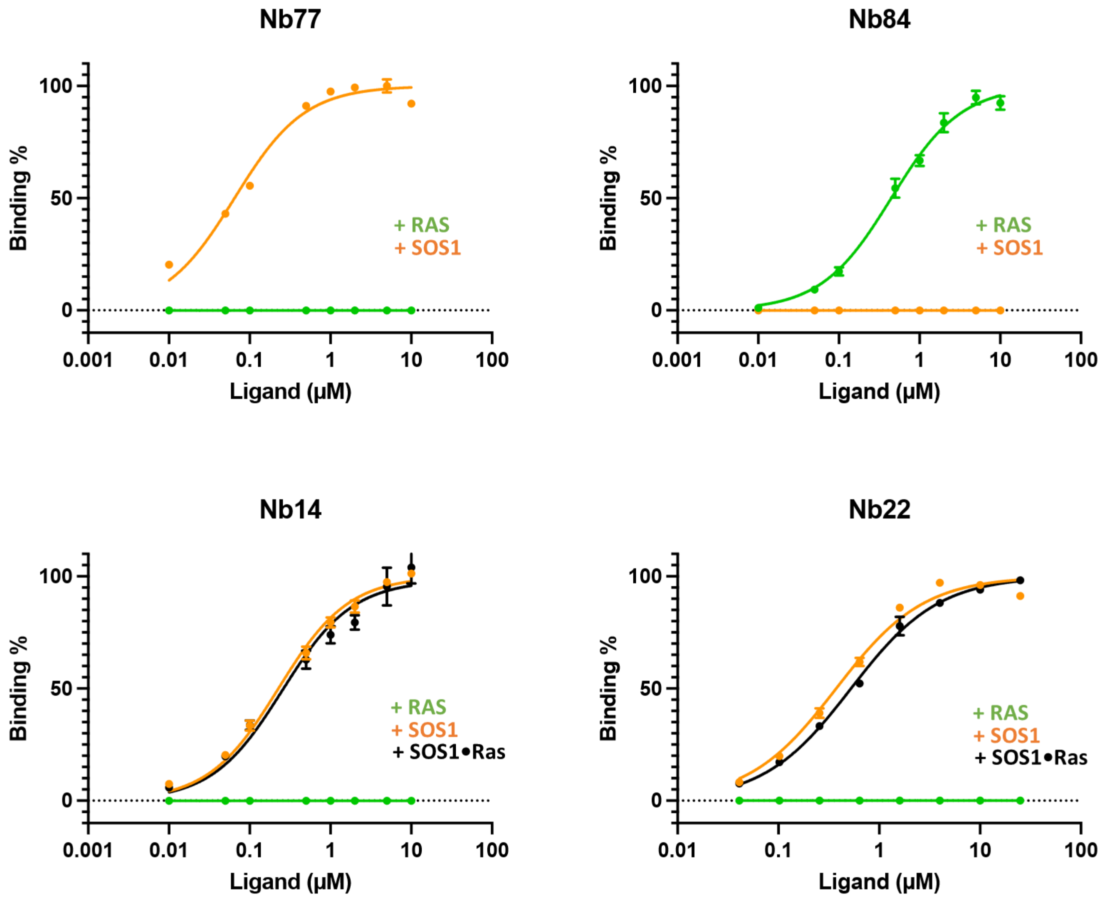

Supplementary Figure 5. Binding affinities of competitive and allosteric nanobodies for SOS1 and RAS analyzed by bio layer interferometry (BLI). Biotinylated Nbs were immobilized on Streptavidin coated biosensors and plunged into solutions containing increasing concentrations of SOS1, RAS or SOS1•RAS. Titration curves were obtained by plotting signal amplitudes of the association step versus antigen concentration to determine affinities. Data are presented as mean values +/- SEM. Nb77 binds SOS1 with an affinity of 0.07 ± 0.01 µM but does not bind RAS. Nb84 binds RAS with an apparent affinity of 0.45 ± 0.03 µM and does not bind SOS1. Nb14 does not bind RAS but binds SOS1 with an apparent affinity of 0.23 ± 0.01 µM and binds the SOS1•RAS complex with an affinity of 0.26 ± 0.03 µM. Nb22 does not bind RAS but binds SOS1 with an affinity of 0.38 ± 0.03 µM and binds the SOS1•RAS complex with an affinity of 0.52 ± 0.02 µM. The experiments were repeated independently three times. Source data are provided as a Source Data file.

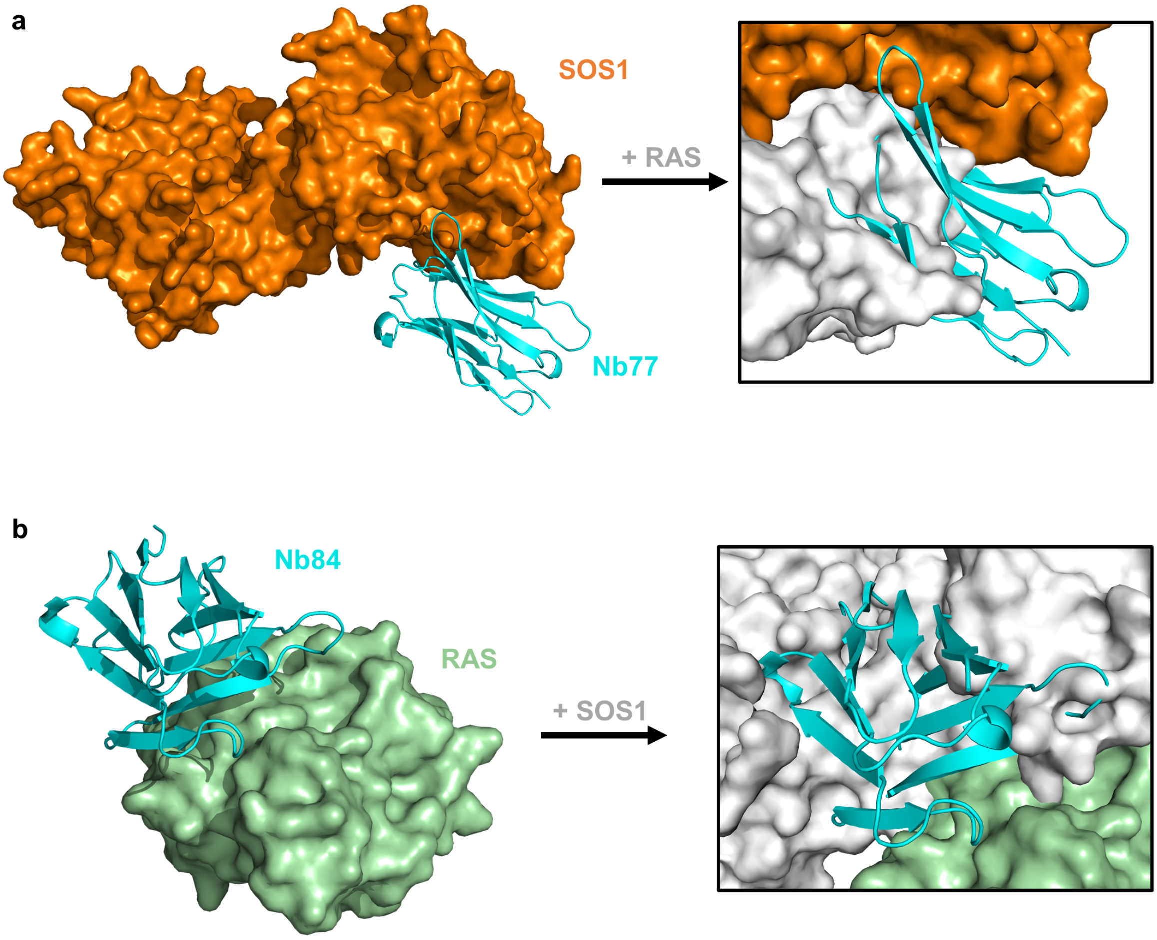

Supplementary Figure 6. Nb77 and Nb84 are competitive binders for RAS (a) and SOS1 (b).

a: The structure of SOS1•Nb77 complex is shown on the left (PDB 8BE2). The overlay of the structures of the SOS1•Nb77 (PDB 8BE2, this study) and SOS1-RAS (PDB 1BKD[1]) complexes reveal steric clashes between Nb77 and RAS.

b. The structure of RAS ($KRAS^{G12V}$•GDP) in complex with Nb84 is shown on the left (PDB 8BE3). The overlay of the structures of the RAS•Nb84 (PDB 8BE3, this study) and SOS1-RAS (PDB 1BKD[1]) complexes reveal steric clashes between Nb84 and SOS1.

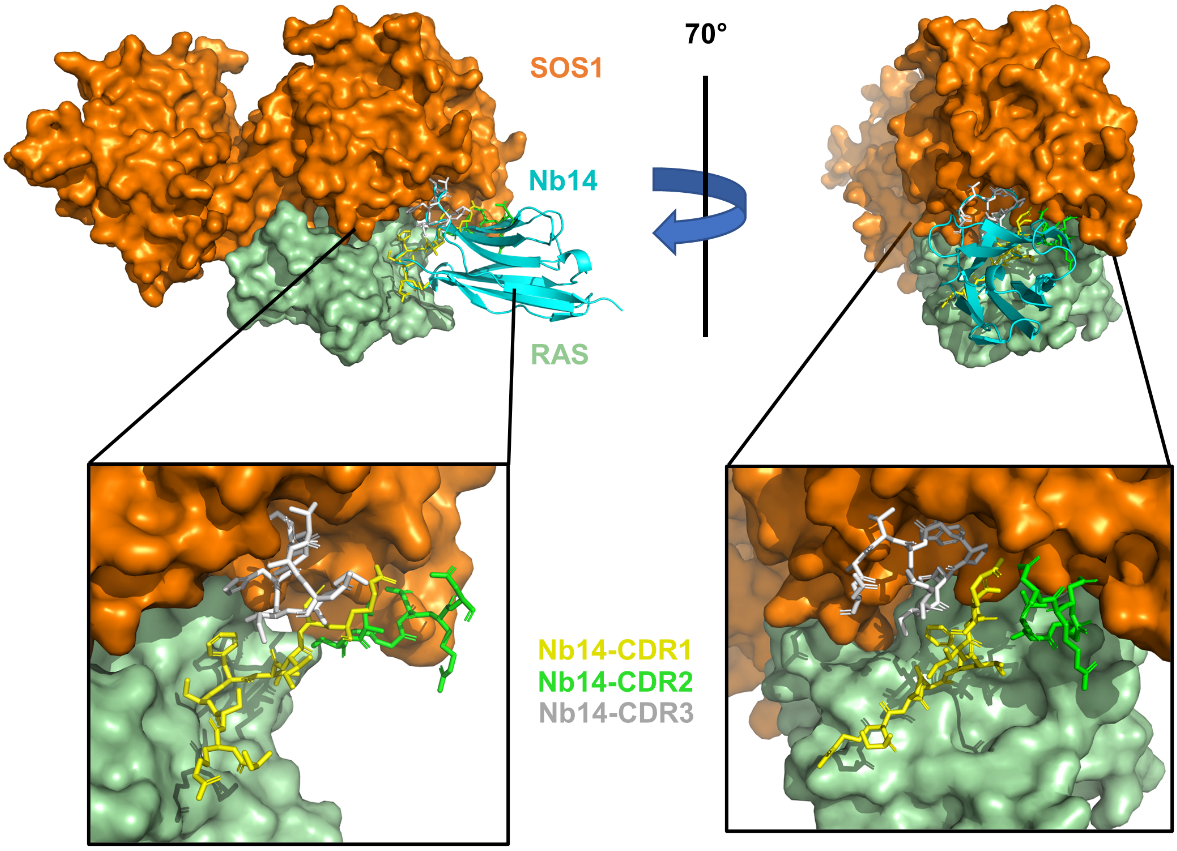

Supplementary Figure 7. Nb14 binds a connective epitope on the SOS1•RAS complex (PDB 8BE4). Residues of the three CDRs (different colors) are represented as sticks. For clarity, the framework residues of Nb14 have been omitted in the zoom-in panels.

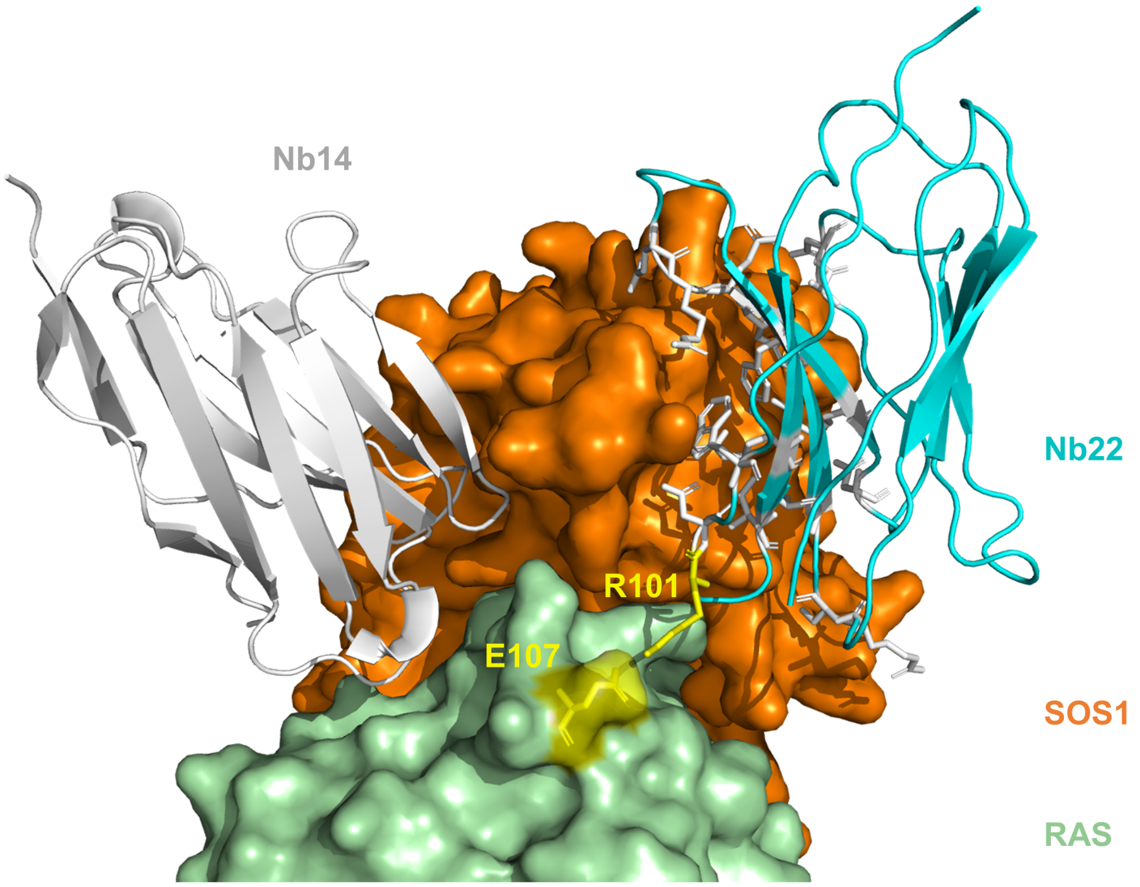

Supplementary Figure 8. Nb22 and Nb14 bind opposite edges of SOS1 in the SOS1•RAS complex (PDB 8BE4 overlayed on PDB 8BE5). Nb22 binds an allosteric site on the Cdc25 domain of SOS1 through framework residues (white sticks). A single arginine residue from Nb22 (R101, yellow sticks) forms a salt bridge with E107 of RAS (yellow sticks).

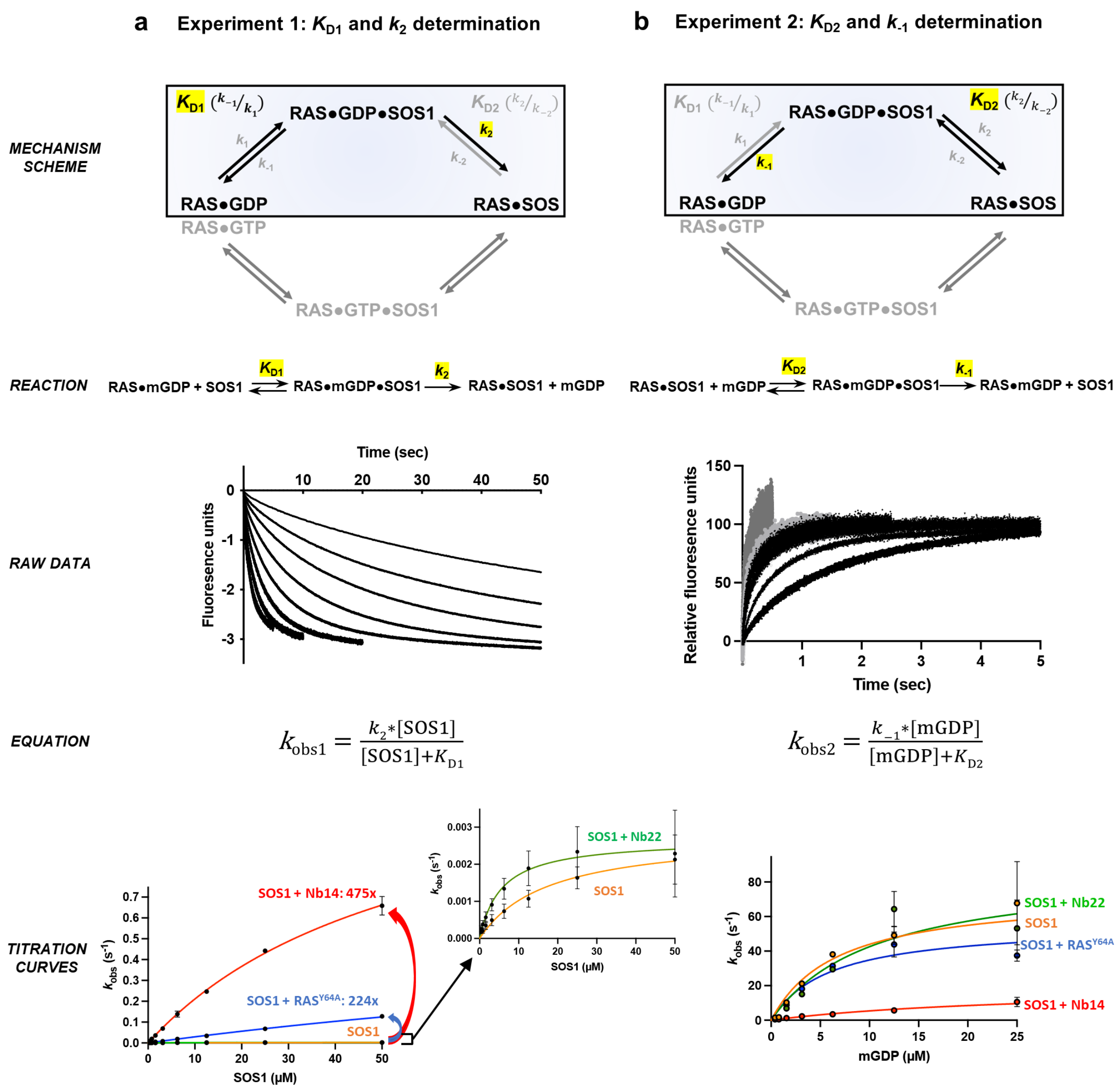

$$k_{obs1} = \frac{k_2 * [SOS1]}{[SOS1] + K_{D1}}$$

$$k_{obs2} = \frac{k_{-1} * [mGDP]}{[mGDP] + K_{D2}}$$

Supplementary Figure 9. Determination of microscopic rate constants of the SOS1-catalyzed nucleotide exchange reaction of RAS in presence of RAS^{Y64A}, Nb14 or Nb22. **a.** K_{D1} and k_2 were obtained by mixing a fixed concentration (0.5 µM) of RAS•mGDP with varying excess concentrations of SOS1 and an excess of GDP. Raw data curves were fitted to a single exponential equation to determine k_{obs1}. Titration curves of k_{obs1} versus the SOS1 concentration were plotted and K_{D1} and k_2 were determined by deriving k_{obs1} with the indicated equation. Data are presented as mean values +/- SEM. A significant increase of k_{obs} and k_2 was observed in presence Nb14 and RAS^{Y64A} (respectively 475x and 224x). For Nb22, k_{obs} and k_2 values were similar to those obtained with SOS1 alone, but were reached with a lower concentration of SOS1, translated into a K_{D1} value decreased by a factor of 3.1. **b.** K_{D2} and k_{-1} were obtained by mixing a fixed concentration (0.5 µM) of SOS1•RAS complex with varying excess concentrations of mGDP. Raw data curves were fitted to a single exponential equation to determine k_{obs2}. Titration curves of k_{obs2} versus the SOS1 concentration were plotted and K_{D2} and k_{-1} were determined by deriving k_{obs2} with the indicated equation. Compared to SOS1, no significant change was observed for Nb22 and RAS^{Y64A}. However, for Nb14 a significant decrease was observed for k_{-1} (0.3x) and a higher concentration of mGDP was required to reach saturation (K_{D2} increased 4x). The experiments were repeated independently three times. Source data are provided as a Source Data file.

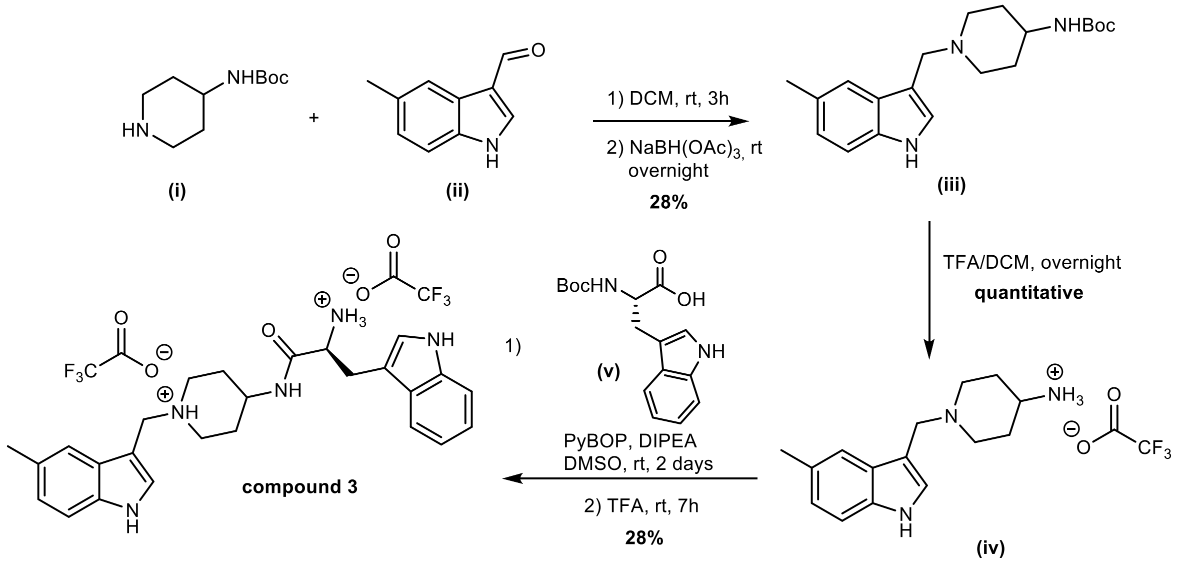

Supplementary Figure 10. Synthesis of the indolo-4-aminopiperidine small molecule modulator (compound 3). M_w = 429.5680 g.mol^{-1}. ^{1}H NMR (250 MHz, DMSO-d_6) δ (ppm) 11.38 (s, 1H), 11.00 (s, 1H), 9.40 (bs, 1H), 8.47 (d, J = 7.5 Hz, 1H), 7.65-6.90 (m, 8H), 4.38 (s, 2H), 3.93-378 (m, 1H), 3.77-3.63 (m, 1H), 3.19-2.94 (m, 3H), 2.41 (s, 3H), 2.00-1.79 (m, 1H), 1.69-1.27 (m, 3H). MS (ESI$^+$) : [M+Na]$^+$ = 452.2150 Da.

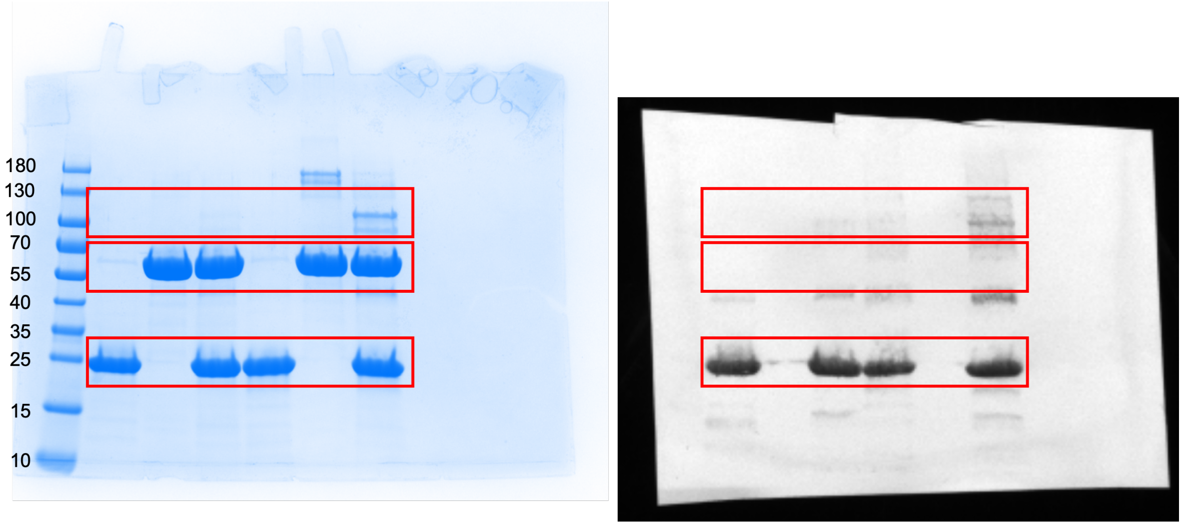

Supplementary Figure 11. Uncropped gel and blot of supplementary Figure 2.

Supplementary Table 1: Crystallographic data collection and refinement statistics

Crystal name (pdb ID)	SOS1-Nb77 (8BE2)	$KRAS^{G12V}$-Nb84 (8BE3)	SOS1-$KRAS^{G12V}$-Nb14 (8BE4)	SOS1-$KRAS^{G12V}$-Nb22-Nb75 (8BE5)
**Data collection**				
Space group	P21 21 21	P1 21 1	I4	I4 2 2
Cell dimensions				
a, *b*, *c* (Å)	80.53, 102.93, 108.49	49.85, 130.50, 55.60	127.67, 127.67, 152.09	124.08, 124.08, 408.82
α, β, γ (°)	90.00, 90.00, 90.00	90.00, 116.25, 90.00	90.00, 90.00, 90.00	90.00, 90.00, 90.00
Resolution (Å)	29.59-1.90 (2.01-1.90)	42.30-1.84 (1.96-1.84)	29.59-1.90 (2.01-1.90)	29.91-3.13 (3.25-3.13)
R_{sym} or R_{merge}	0.07 (1.19)	0.08 (1.06)	0.07 (0.86)	0.14 (1.16)
$I / \sigma I$	14.38 (1.53)	10.73 (1.36)	15.63 (1.82)	13.65 (1.76)
Completeness (%)	99.8 (99.1)	98.6 (94.7)	99.6 (97.5)	99.3 (97.0)
Redundancy	6.95 (6.86)	4.72 (4.63)	6.89 (6.84)	10.82 (10.45)
**Refinement**				
Resolution (Å)	29.59-1.90 (1.97-1.90)	42.30-1.84 (1.91-1.84)	29.59-1.90 (1.96-1.90)	29.91-3.13 (3.25-3.13)
No. reflections	71637	53897	95496	28506
R_{work} / R_{free}	0.182 / 0.211	0.201 / 0.230	0.175 / 0.198	0.226 / 0.278
No. atoms	5121	4725	6622	6443
Protein	4687	4373	6071	6424
Ligand/ion	25	2	0	0
Water	409	350	551	19
B-factors	43.6	41.2	41.7	93.6
Protein	43.0	41.1	41.2	93.6
Ligand/ion	78.8	29.0		
Water	48.4	44.1	47.3	79.64
R.m.s. deviations				
Bond lengths (Å)	0.01	0.01	0.02	0.01
Bond angles (°)	0.82	0.75	1.39	1.38

*Values in parentheses are for highest-resolution shell.

Supplementary references

1. Boriack-Sjodin, P.A., Margarit, S.M., Bar-Sagi, D. & Kuriyan, J. The structural basis of the activation of Ras by Sos. *Nature* **394**, 337-343 (1998).
