# Supplementary material for: Allosteric nanobodies to study the interactions between SOS1 and RAS
Source: Nat Commun. 2024 Jul 23;15:6214. doi: 10.1038/s41467-024-50349-2 (PMC11266648; doi:10.1038/s41467-024-50349-2)
Supplement: Supplementary file 3 — Reporting Summary [file 41467_2024_50349_MOESM3_ESM.pdf]

nature portfolio

Corresponding author(s): Jan Steyaert

Last updated by author(s): Apr 15, 2024

Reporting Summary

Nature Portfolio wishes to improve the reproducibility of the work that we publish. This form provides structure for consistency and transparency in reporting. For further information on Nature Portfolio policies, see our Editorial Policies and the Editorial Policy Checklist.

Statistics

For all statistical analyses, confirm that the following items are present in the figure legend, table legend, main text, or Methods section.

n/a	Confirmed	
☐	☒	The exact sample size (*n*) for each experimental group/condition, given as a discrete number and unit of measurement
☐	☒	A statement on whether measurements were taken from distinct samples or whether the same sample was measured repeatedly
☒	☐	The statistical test(s) used AND whether they are one- or two-sided<br>*Only common tests should be described solely by name; describe more complex techniques in the Methods section.*
☒	☐	A description of all covariates tested
☒	☐	A description of any assumptions or corrections, such as tests of normality and adjustment for multiple comparisons
☐	☒	A full description of the statistical parameters including central tendency (e.g. means) or other basic estimates (e.g. regression coefficient) AND variation (e.g. standard deviation) or associated estimates of uncertainty (e.g. confidence intervals)
☒	☐	For null hypothesis testing, the test statistic (e.g. *F*, *t*, *r*) with confidence intervals, effect sizes, degrees of freedom and *P* value noted<br>*Give P values as exact values whenever suitable.*
☒	☐	For Bayesian analysis, information on the choice of priors and Markov chain Monte Carlo settings
☒	☐	For hierarchical and complex designs, identification of the appropriate level for tests and full reporting of outcomes
☒	☐	Estimates of effect sizes (e.g. Cohen's *d*, Pearson's *r*), indicating how they were calculated

Our web collection on statistics for biologists contains articles on many of the points above.

Software and code

Policy information about availability of computer code

Data collection	Proxima 1 beamline of the Soleil synchrotron (Saint-Aubin, France) and I03 & I24 beamlines at the Diamond Light Source synchrotron (Oxfordshire, UK) for X-ray diffraction data<br>Bruker Avance III HD 800 MHz spectrometer for NMR data acquisition<br>microplate reader (Tecan) and stopped-flow apparatus (Applied Photophysics) for fluorescence measurements<br>Octet Red96 (Forte Bio, Inc.) for BLI measurements<br>FACS Fortessa (BD Biosciences) for flow cytometry
Data analysis	XDS v. June 1, 2017, for indexing and integrating X-ray diffraction data<br>COOT and PHENIX suite for structure determination and refinement<br>Molprobity v 4.2 for analysis and validation of sterics and geometry of protein crystal structrues<br>PyMOL v. 2.5.0 for visualizing 3D molecular structures and creation of high quality figures<br>FlowJo software (FlowJo, LLC) for flow cytometry analysis<br>GraphPad Prism v 9.4.1 for kinetic data<br>Compass for SW (version 3.1.8, Biotechne) for protein level quantification<br>TopSpin 3.6 (Bruker) for recording, processing and analysis of NMR data

For manuscripts utilizing custom algorithms or software that are central to the research but not yet described in published literature, software must be made available to editors and reviewers. We strongly encourage code deposition in a community repository (e.g. GitHub). See the Nature Portfolio guidelines for submitting code & software for further information.

Data

Policy information about availability of data

All manuscripts must include a data availability statement. This statement should provide the following information, where applicable:

- Accession codes, unique identifiers, or web links for publicly available datasets
- A description of any restrictions on data availability
- For clinical datasets or third party data, please ensure that the statement adheres to our policy

Crystal structures of SOS1-Nb77, KRasG12V-Nb84, SOS1-KRasG12V-Nb14 and SOS1-KRasG12V-Nb75-Nb22 have been deposited in the Protein Databank (PDB) with their respective diffraction data under accession codes 8BE2, 8BE3, 8BE4 and 8BE5, respectively.

Research involving human participants, their data, or biological material

Policy information about studies with human participants or human data. See also policy information about sex, gender (identity/presentation), and sexual orientation and race, ethnicity and racism.

Reporting on sex and gender	N/A
Reporting on race, ethnicity, or other socially relevant groupings	N/A
Population characteristics	N/A
Recruitment	N/A
Ethics oversight	N/A

Note that full information on the approval of the study protocol must also be provided in the manuscript.

Field-specific reporting

Please select the one below that is the best fit for your research. If you are not sure, read the appropriate sections before making your selection.

☒ Life sciences ☐ Behavioural & social sciences ☐ Ecological, evolutionary & environmental sciences

For a reference copy of the document with all sections, see nature.com/documents/nr-reporting-summary-flat.pdf

Life sciences study design

All studies must disclose on these points even when the disclosure is negative.

Sample size	No sample size calculation was performed<br>Where biochemical dose response effects, or bacterial growth are reported, these are reported as means ±SD of at least three biological (i.e. independent) replicates, as is standard in the field. Sample sizes for each experiment are described in the legend of the respective figures.
Data exclusions	No data were excluded
Replication	Each of the kinetic measurements (fluorescence and BLI) was carried out at least in duplicate and plotting measured data (mean ± standard deviation) vs. X-axis yielded curves to obtain values ± standard error. All attempts at repetition were successfull<br>For cellular experiments, three independent replicates (i.e. independent innocula starting from untransfected cells) were performed and used in the data analysis and presenation<br>All NMR experiments were performed in duplicates or triplicates and all attempts at repetition were successfull
Randomization	Randomization was not relevant to our study, as we did not compare groups.
Blinding	Blinding was not applicable as we used predetermined samples, conditions and time points in our assays

Reporting for specific materials, systems and methods

We require information from authors about some types of materials, experimental systems and methods used in many studies. Here, indicate whether each material, system or method listed is relevant to your study. If you are not sure if a list item applies to your research, read the appropriate section before selecting a response.

Materials & experimental systems

n/a	Involved in the study
☐	☒ Antibodies
☐	☒ Eukaryotic cell lines
☒	☐ Palaeontology and archaeology
☒	☐ Animals and other organisms
☒	☐ Clinical data
☒	☐ Dual use research of concern
☒	☐ Plants

Methods

n/a	Involved in the study
☒	☐ ChIP-seq
☐	☒ Flow cytometry
☒	☐ MRI-based neuroimaging

Antibodies

Antibodies used

All Nanobodies DNA constructs are available upon request.
Rabbit polyclonal phospho-ERK1/2 (1:50, Cell Signaling #9101)
Mouse monoclonal ERK1/2 (1:50, clone L34F12, Cell Signaling #4696)
Mouse monoclonal Flag antibody (clone M2, 1:1000, Merck)
Anti-rabbit secondary HRP antibody (ready-to-use, Biotechne (ProteinSimple), # 042-206)
Anti-mouse secondary HRP antibody (1:50, Cytiva, NA931-1ML)

Validation

Nanobodies Nb77, Nb84, Nb14 and Nb22 were validated by bio-layer interferometry
All antibodies were validated by the manufacturers with the Western Blot analysis as shown in the website of the manufacturer.

Eukaryotic cell lines

Policy information about cell lines and Sex and Gender in Research

Cell line source(s)

EBY100 yeast strain: received from K.D. Wittrup (Massachusetts Institute of Technology, Cambridge, US)
HEK293

Authentication

None of the cell lines were further authenticated.

Mycoplasma contamination

All cell lines were tested and were negative for the mycoplasma (Lonza MycoAlert Mycoplasma Detection Kit).

Commonly misidentified lines
(See ICLAC register)

No commonly misidentified cell line was used.

Plants

Seed stocks

Report on the source of all seed stocks or other plant material used. If applicable, state the seed stock centre and catalogue number. If plant specimens were collected from the field, describe the collection location, date and sampling procedures.

Novel plant genotypes

Describe the methods by which all novel plant genotypes were produced. This includes those generated by transgenic approaches, gene editing, chemical/radiation-based mutagenesis and hybridization. For transgenic lines, describe the transformation method, the number of independent lines analyzed and the generation upon which experiments were performed. For gene-edited lines, describe the editor used, the endogenous sequence targeted for editing, the targeting guide RNA sequence (if applicable) and how the editor was applied.

Authentication

Describe any authentication procedures for each seed stock used or novel genotype generated. Describe any experiments used to assess the effect of a mutation and, where applicable, how potential secondary effects (e.g. second site T-DNA insertions, mosiacism, off-target gene editing) were examined.

Flow Cytometry

Plots

Confirm that:

☒ The axis labels state the marker and fluorochrome used (e.g. CD4-FITC).

☒ The axis scales are clearly visible. Include numbers along axes only for bottom left plot of group (a 'group' is an analysis of identical markers).

☒ All plots are contour plots with outliers or pseudocolor plots.

☒ A numerical value for number of cells or percentage (with statistics) is provided.

Methodology

Sample preparation

Yeast display experiments were performed using EBY100 yeast cells transformed with DNA plasmids as indicated in methods.

Sample preparation	Yeast cells displaying a nanobody library or individual nanobody variants were routinely inoculated, induced and orthogonally stained with CoA derivatives (CoA-488 or CoA-647) to monitor the display level of the Nb-Aga2P-ACP-fusion on each yeast cell as described (https://doi.org/10.1038/s41598-018-37212-3). Next, stained yeast cells were incubated with fluorescent, cognate antigens as indicated in methods, and subjected to fluorescence-activated cell sorting (BD FACS Aria) or flow-cytometryc analysis (BD FACS Fortessa)
Instrument	BD FACS Aria, BD FACS Fortessa
Software	BD DivaFACS v8.0, LLC FLowJo v10
Cell population abundance	The abundance of selected clones was indicated by the increased enrichment of positive cells within subsequence rounds of selection procedure and additionally validated by sequencing and FACS screen of individual clones
Gating strategy	Gating strategy is described in Supplementary Data Figure 3a.

☒ Tick this box to confirm that a figure exemplifying the gating strategy is provided in the Supplementary Information.
